# Pharmacological mTOR inhibitors in ameliorating Alzheimer’s disease: current review and perspectives

**DOI:** 10.3389/fphar.2024.1366061

**Published:** 2024-05-30

**Authors:** Pei-Lun Xie, Meng-Yu Zheng, Ran Han, Wei-Xin Chen, Jin-Hua Mao

**Affiliations:** ^1^ University College London, London, United Kingdom; ^2^ King’s College London, London, United Kingdom; ^3^ Dongfang Hospital of Beijing University of Chinese Medicine, Beijing, China; ^4^ Beijing University of Chinese Medicine, Beijing, China

**Keywords:** Alzheimer’s disease, mTOR, rapamycin, autophagy, neuroinflammation, neurovascular system

## Abstract

Traditionally, pharmacological mammalian/mechanistic targets of rapamycin (mTOR) kinase inhibitors have been used during transplantation and tumor treatment. Emerging pre-clinical evidence from the last decade displayed the surprising effectiveness of mTOR inhibitors in ameliorating Alzheimer’s Disease (AD), a common neurodegenerative disorder characterized by progressive cognitive function decline and memory loss. Research shows mTOR activation as an early event in AD development, and inhibiting mTOR may promote the resolution of many hallmarks of Alzheimer’s. Aberrant protein aggregation, including amyloid-beta (Aβ) deposition and tau filaments, and cognitive defects, are reversed upon mTOR inhibition. A closer inspection of the evidence highlighted a temporal dependence and a hallmark-specific nature of such beneficial effects. Time of administration relative to disease progression, and a maintenance of a functional lysosomal system, could modulate its effectiveness. Moreover, mTOR inhibition also exerts distinct effects between neurons, glial cells, and endothelial cells. Different pharmacological properties of the inhibitors also produce different effects based on different blood-brain barrier (BBB) entry capacities and mTOR inhibition sites. This questions the effectiveness of mTOR inhibition as a viable AD intervention strategy. In this review, we first summarize the different mTOR inhibitors available and their characteristics. We then comprehensively update and discuss the pre-clinical results of mTOR inhibition to resolve many of the hallmarks of AD. Key pathologies discussed include Aβ deposition, tauopathies, aberrant neuroinflammation, and neurovascular system breakdowns.

## 1 Introduction

Alzheimer’s disease (AD) is a complex neurodegenerative disorder and the leading cause of dementia in the elderly. The etiology of AD is uncertain and likely multifactorial. Age represents the primary risk factor for AD, with most patients above 65 years old, although AD has also been associated with other conditions such as head traumas, infections, diabetes, and environmental factors (Breijyeh and Karaman, 2020; Bellenguez et al., 2022). Classic hallmarks of the disease include amyloid-beta (Aβ) plaque deposition, tau protein hyperphosphorylation, and neurofilament tangle formation (NFT). In addition, patients with AD exhibit diverse histopathological phenotypes such as increased oxidative damage, xenobiotic invasion, neuroinflammation, and compromised neurovascular functions. These are thought to lead to AD phenotypes including learning defects, memory loss, seizures, and death. Globally, over 50 million patients are currently diagnosed with AD, which is expected to grow to 153 million in 2050 (GBD, 2019 Dementia Forecasting Collaborators, 2022). AD is also a significant contributor to healthcare costs, contributing to a total toll of 1.3 trillion USD in dementia-associated spendings.

Accumulating evidence demonstrates the role of the mammalian/mechanistic target of rapamycin (mTOR) pathway in neurological diseases including AD. mTOR is a protein kinase that maintains energy homeostasis and protein synthesis in various tissues of the body, including the brain (Hoeffer and Klann, 2010). mTOR is an intracellular serine/threonine kinase with a carboxyl terminal catalytic domain. mTOR is the catalytic subunit of two different signaling complexes, mTOR Complex 1 (mTORC1) and mTOR Complex 2 (mTORC2). mTOR associates with Raptor, PRAS40, mlST8 to form mTORC1, and associates with RICTOR, PROTOR, mSIN1, mLST8, and DEPTOR in mTORC2 (H. Yang et al., 2013). mTORC1 and mTORC2 have been reported in diverse subcellular locations, including the plasma membrane, lysosome, mitochondria, cytoplasm, stress granules, and nucleus (Betz and Hall, 2013), although the precise role of mTOR complexes’ location in neurodegenerative disorders remains less clear. Activated on the lysosome membrane by the GTP-binding protein Rheb, the mTOR complexes may then integrate information from distinct intracellular and environmental factors, including growth hormones, energetic stress, and oxygen availability. In turn, they regulate proteostasis and autophagy, mitochondrial dynamics, cell growth and proliferation, and the hypoxia response (Saxton and Sabatini, 2017). In the central nervous system (CNS), mTOR and associated downstream factors are involved in synaptic plasticity, neuroendocrine regulation, microglia activation, neurovascular coupling, and memory retention. mTOR and associated pathway mutations lead to multiple cancers, metabolic syndrome, and neurodegenerative diseases. mTORC1 hyperactivation has been associated with autism, epilepsy, seizures, and the progression of Alzheimer’s and Parkinson’s Disease (Perluigi et al., 2015; Pagani et al., 2021; Gourmaud et al., 2022).

Arguments have thus been made discussing the relevance of mTOR-based interventions in future AD treatments, with varied opinions (Carosi and Sargeant, 2019; 2023; Kaeberlein and Galvan, 2019; Panwar et al., 2023). Representative of these arguments are those presented by Kaeberlein et al. and Carosi et al. Briefly, Kaeberlein et al. argue for the clinical testing of mTOR inhibitors as an intervention strategy, coming from the observation of abnormal mTOR activation in a multitude of neurodegenerative disorders, coupled with the predominantly positive pre-clinical evidence of mTOR inhibitors in resolving many AD-associated pathologies (Spilman et al., 2010; Lin et al., 2013; Siman et al., 2015). Yet, AD animal models are different from patient phenotypes. Moreover, pathologies of Alzheimer’s and the body’s responses to AD shift pronouncedly with disease progression. Carosi et al. critically points out the time-sensitive and model-dependent nature of these pre-clinical successes to urge caution (Majumder et al., 2011; Carosi and Sargeant, 2023). Recent evidence adds another layer of complexity to mTOR inhibitor-based interventions. Neuroinflammation, a characteristic traditionally thought to worsen AD pathology, may be required for early-stage AD amelioration (Leng and Edison, 2021). mTOR inhibitors are known to regulate neuroinflammation in diverse brain cell types, but have largely been overlooked in the context of AD development. A final point relates to the diversity of mTOR inhibitors. With diverse pharmacological properties, it has been difficult to interpret and compare experimental outcomes without insights into their functional differences.

Thus, this review will first present the mechanisms of action of existing mTOR inhibitors and consider their safe clinical applicability in humans. The review then discusses the current pre-clinical findings of mTOR complexes in Alzheimer’s pathologies and provides an up-to-date assessment of the use of mTOR inhibitors in AD, in particular consideration of the different viewpoints. We highlight treatment outcomes for a variety of AD-associated symptoms from different model systems, aiming to promote the evaluation of the translational potential of mTOR inhibitors and guide future research.

## 2 Properties of mTOR inhibitors

There has been an increasing effort to develop the number and categories of mTOR inhibitors with varying pharmacological properties, stemming from their use for organ transplant immunosuppression and inhibition of cancer cell proliferation (Benjamin et al., 2011; Mao et al., 2022). However, different mTOR inhibitors may have significantly different properties in mTOR inhibition. They can be sorted into three broad categories according to their clinical relevance. 1) Rapamycin. 2) Rapalogs. 3)ATP-competitive mTOR inhibitors. Other novel mTOR inhibitors or dual inhibitors are also described.

### 2.1 Rapamycin

Rapamycin (Sirolimus) is a macrolide and the first discovered mTOR kinase inhibitor. It remains the most applied agent to inhibit the mTORC1 complex in pre-clinical and clinical models due to extensive research backgrounds. Rapamycin was originally discovered on the island of Rapa Nui in 1972 from *Streptomyces hygroscopicus* (Heitman et al., 1991)*.* It became noted for its immunomodulatory effects to restrict T-cell and B-cell proliferation, although later research demonstrate anti-inflammatory properties in the innate immune system by controlling myeloid cell phagocytosis, chemotaxis, proliferation and survival, and cytokine production (Weichhart et al., 2015). It has gained FDA approval in the United States in 1999 against hyperacute and acute graft rejection in kidney transplants (Saunders et al., 2001), in 2003 as an antirestenosis agent to prevent post-angioplasty coronary artery estenosis, and as an advanced metastatic tumor treatment in 2021 (Hua et al., 2019). Clinically, Rapamycin has often been applied as part of drug cocktail in treatment. Rapamycin has also been tested in clinical trials in replasing-remitting multiple sclerosis and amyloidtrophic lateral sclerosis patients with anti-inflammatory effects reported (Salehi et al., 2016; Mandrioli et al., 2023). No Alzheimer’s disease clinical trial applying rapamycin has been completed to date, two although a phase IIa clinical trials are set to be completed in 2024 and 2025, respectively (Svensson et al., 2023; Hou et al., 2024). Both studies will examine rapamycin treatment in mild cognitive impairment (MCI) staged patients, with Svenssen et al. employing positron emission tomography and magnetic resonance imaging to investigate cerebral glucose uptake and cognitive markers to evaluate outcome (Svensson et al., 2023). Other effects of the drug include pro-longevity effects, possibly via enhanced protein aggregate clearance, stem cell maintenance, mitochondrial dynamics control, and inflammation reduction.

Rapamycin’s inhibitory activities on mTORC1 has been well described (Choi et al., 1996). Rapamycin is frequently administered via intravenal delivery and requires prior solubilization given its highly hydrophobic property. This hydrophobicity is essential to its binding to mTOR and an associated immunophilin protein family termed FK506-binding protein (FKBP). Rapamycin can bind to different FKBPs to form a gain-of-function complex that allosterically inhibits the mTOR kinase. Mechanistically, Rapamycin binds to two hydrophobic pockets located on the mTOR kinase (FKBP-rapamycin binding domain; FRB) and two hydrophobic pockets of the associated FKBP, forming a heterodimeric complex. mTOR kinase activity is inhibited by the associated FKBP, which sterically restricts substrate serine/threonine entry into the kinase catalytic cleft, inhibiting phosphorylation. Rapamycin does not restrict all mTORC1 kinase substrate entry. For instance, 4E-BP1 activation is weakly elevated by rapamycin, and additional 4E-BP1 inhibition has been shown to contribute to maximal *in vitro* cancer suppression (Choo et al., 2008).

### 2.2 Novel rapamycin interacting partners

Rapamycin interacts with a range of non-mTORC1 molecular partners. Notably, rapamycin inhibits mTORC2 in a FKBP-dependent manner. An increased FKBP12 ratio has been associated with increased mTORC2 activation. Furthermore, FKBP12 and FKBP51 knockdowns can respectively impair and sensitize rapamycin-mediated inhibition of mTORC2 (Schreiber et al., 2015). Traditionally, it has been thought that rapamycin exclusively inhibits the mTOR kinase in mTORC1, and that mTORC2 function is not inhibited by rapamycin. Importantly, the emerging functional importance of mTORC2 in AD progression requires better understanding of the rapamycin-mTORC2 interaction. A study in the prefrontal and temporal cortex of late-staged (Braak’s stage V-VI) Alzheimer’s patients show that activation phosphorylation (Thr1135) of Rictor, an mTORC2 activator protein, negatively correlated with Alzheimer’s progression (H. K. Lee et al., 2017). This was replicated in the APPsw Aβ mice model neurons and Aβ42-exposed SH-SY5Y neuronal cells (H. K. Lee et al., 2017). It has been noted that some post-mortem studies have revealed contrasting results. Sun et al. show that Rictor expression levels are unchanged in the AD brain hippocampus relative to healthy individuals, suggesting that mTORC2 is not upregulated (Sun et al., 2014). Rictor Thr1135 phosphorylation levels were not assessed. Nonetheless, it is possible that Rapamycin can influence AD in a mTORC2-dependent manner through its binding interactions, necessitating investigation.

Although rapamycin has also been implicated in inhibiting other enzymes outside of the mTOR kinase, it has generally been thought to not play a role in its pharmacology. Recent findings support this assumption and highlight rapamycin’s efficacy. It was known that rapamycin can displace FKBP1b binding activity with Ryanodine receptors (RyR) to inhibit calcium ion release and electrophysiological processes (Kaftan et al., 1996). It may also inhibits the transient receptor potential mucolipin 1 (TRPML1), a [PI(3,5)P2]–gated lysosomal cation channel that promotes autophagy (Gan et al., 2022). However, it is important to note that these occurred at much higher concentrations than rapamycin’s *in vivo* availability. One recent analyses shown that these inhibitory effect were only observed micromolar concentration, as opposed to the than the standard nanomolar rapamycin levels administered to AD models (Artoni et al., 2023). Rapamycin displays extremely high selectivity for the mTOR kinase at lower concentrations, and it will be interesting to assess whether this is replicated under Alzheimer’s settings.

### 2.3 Rapalogs

Rapalogs are second generation mTOR inhibitors developed in response to the low solubility and poor pharmacokinetics of rapamycin. Rapalogs are structurally similar to rapamycin and similarly recruit mTOR and FKBP proteins to maintain inhibition (Le Tourneau et al., 2008). Therefore, the interacting partners of rapamycin and rapalogs are thought to be similar. Different rapalogs are characterised by different modifications on the C42 or C40 functional groups to enhance its pharmacological properties. Examples of which include temsirolimus, everolimus, and ridaforolimus (Mabasa and Ensom, 2005; Bukowski, 2012; Mita et al., 2013). For instance, temsirolimus, an ester derivative of the rapamycin, displays better stability and solubility. *In vitro*, it is similar to rapamycin in terms of potency and mTOR signaling inhibition effectiveness (Maki et al., 2007; Silk and Schuetze, 2012). Some evidence shows different interactions between the two mTOR inhibitors in selectivity. Everolimus has been reported to display greater mTORC2 interacting potency and greater effectiveness in reducing neuroinflammation in *in vitro* neuron models (M. T. Yang et al., 2017), which would benefit from in-depth mechanistic evaluation. Neither first generation nor second generation mTOR inhibitors may effectively cross the blood-brain barrier (BBB), a selective structure that meditates substrate exchange between cerebral interstitial fluids and the microvasculature (Mao et al., 2022).

### 2.4 Small molecule mTOR inhibitors

Third generation mTOR inhibitors are small hydrophobic compounds that competitively bind to the mTOR kinase ATP binding pocket to achieve inhibition. Molecularly, small molecule inhibitors are structurally different to rapamycin and rapalogs and does not rely on FKBP cooperation. Examples include PP242, Torin1, torin2, and PP30 (Tramutola et al., 2017). Functionally, unlike rapamycin and rapalogs, small molecule inhibitors display strong inhibitory binding to both the mTOR catalytic pockets in mTORC1 and mTORC2 (Mao et al., 2022). Indeed, these ATP-competitive mTOR inhibitors display more complete mTOR kinase inhibition in comparison to allosteric inhibitors, possibly contributing to (T. Xu et al., 2020). Furthermore, given their small hydrophobic properties, ATP-competitive mTOR inhibitors are significantly more brain-permeable. In the clinic, this group of mTOR inhibitors have largely been applied in cancer treatments as anti-proliferative and anti-growth drugs with an acceptable toxicity profile (Rodrik-Outmezguine et al., 2016). Given the homology between mTOR and Pi3K, an upstream kinase activator of mTOR, small molecules with mTOR/Pi3K dual-binding site inhibitions have also been developed. These drugs exhibit greater inhibition via inhibiting multiple interactors of the mTOR axis. Examples include PQR530, PI-103, NVPBEZ235, XL765, and more (Benjamin et al., 2011; Tramutola et al., 2017). Current developments of mTOR inhibitors focus on rapalink-1, which contains a linker regions that joins the ATP competitive mTOR inhibitor MLN0128 with rapamycin to promote stronger mTOR suppression (Rodrik-Outmezguine et al., 2016).

## 3 mTOR inhibition in Alzheimer’s models

### 3.1 mTOR inhibition and amyloid-beta pathology

#### 3.1.1 mTOR and amyloid-beta synthesis in Alzheimer’s

Amyloid-Beta (Aβ) is a crucial biomarker in AD, with its accumulation being a central feature in disease progression. Initially found in the neocortex of pre-clinical AD patients, Aβ plaques spread to regions including the hippocampus and basal ganglia in later stages, and eventually to the cerebellar cortex and lower brain stem in advanced cases (Kepp et al., 2023). Some amyloid imaging studies indicate the presence of amyloid deposits in many individuals without clinical symptoms, and conversely, the absence of significant amyloid deposits in some AD patients. However, despite these variations, it is generally agreed that Aβ is a major drive of AD, and that the accumulation of Aβ amyloid fibrils can evolve into senile plaques. These plaques are implicated in neurotoxicity and the initiation of tau pathology, which in turn leads to the death of neuronal cells and neurodegeneration characteristic of AD.

The amyloid cascade hypothesis, a prominent theory in AD research, posits that the pathological production and accumulation of Aβ peptides are central to the disease’s pathogenesis. This hypothesis continues to guide much of the global research and therapeutic approaches for AD. In the context of AD, there is an aberrant increase in the cleavage of β-amyloid precursor protein (APP) by β-secretases, followed by γ-secretases, leading to the generation of Aβ peptides of differing lengths. This process involves sequential enzymatic cleavages by β- and γ-secretases. Notably, the cleavage site of another enzyme involved in APP processing, α-secretase, exists within the Aβ sequence, thereby preventing the formation of Aβ. It is hypothesized that the longer forms of Aβ, such as Aβ42, are more pathogenic due to their increased hydrophobic nature, propensity for oligomerization, and tendency to form amyloid plaques. (Tramutola et al., 2015; Tiwari et al., 2019; Kepp et al., 2023). Several studies have associated increased Aβ42 production with increased oxidative stress and autophagy impairment to produce proteostasis dysregulation in the brain (Di Domenico et al., 2013; Tramutola et al., 2015). Moreover, it may block synaptic transmission, increase neuroinflammation, trigger neuronal cell death, thereby contributing to the pathology of AD.

mTOR activation is commonly associated with AD progression and worsened amyloidogenic Aβ synthesis and deposition. *Postmortem* biopsies from AD patients have shown heightened activating phosphorylation of mTOR, increased phosphorylation of mTOR downstream targets (4E-BP1 and S6K) (X. Li et al., 2005; Tramutola et al., 2015), as well as hyperactivity of the phosphoinositide 3-kinase (PI3K)/Akt/mTOR phosphorylation cascade. Brain tissues diagnosed with mild cognitive impairment (MCI) and AD show higher mTOR activating phosphorylation than pre-clinical AD. This demonstrates a correlation between mTOR activity and disease severity as marked by Braak’s staging. Recent analyses on general population and AD patient GWAS data concerning (PI3K)/Akt/mTOR pathway proteins (Akt, S6K, eIF4E) suggest that genetic backgrounds with elevated mTOR-dependent response is causally linked to AD incidences (H. Y. Cai et al., 2023).

Recent studies have highlighted the role of mTOR in modulating Aβ levels through the regulation of autophagy. This is substantiated by *postmortem* biopsies, which reveal diminished autophagy in AD and MCI patients. This reduction is linked to mTOR-mediated inhibition of key autophagy regulators, such as unc-51-like kinase 1 (ULK1) and transcription factor EB (TFEB) (H. Y. Cai et al., 2023). ULK1 is essential to autophagy initiation and autophagosome formation, and TFEB is a master regulator of lysosomal biogenesis. Autophagosomes engulf cellular components targeted for removal, then fuse with lysosomes for hydrolytic degradation. Quantitative evidence shows a decrease of at least 25% in autophagy markers LC-3 and Beclin one in these patients, indicating significant autophagy impairments. Corroborating this, previous studies have identified a strong correlation between elevated concentrations of Aβ, particularly Aβ42, and pronounced reductions in autophagy across all clinical groups (Tiwari et al., 2019). Suelves et al. further support this association by demonstrating intraneuronal Aβ accumulation as a consequence of autophagic defects in both AD patients and the 5xFAD AD mouse model (Suelves et al., 2023). Complementing these findings, Caccamo et al. observed increased S6K phosphorylation in the 7PA2 immortalized cell line transfected with mutant amyloid precursor protein (APP), and in the brains of 3xTg-AD model mice. Likewise, this mTOR activation exerted pro-disease progression effects via autophagy inhibition (Caccamo et al., 2010).

Interestingly, genetically preventing Aβ accumulation effectively reduced mTOR activity (Caccamo et al., 2011) and upregulated autophagy. Primary cortical neurons treated with Aβ oligomers corroborate these results and indicate that Aβ can increase levels of p- Akt^S473^ and phospho-mTOR^s2448^ (Bhaskar et al., 2009), suggesting a cascade-like increase in Aβ production. Moreover, when naturally secreted Aβ was injected into the hippocampus of wild-type mice, it is capable of enhancing mTOR signalling in their brains via proline-rich Akt substrate 40 (PRAS40) (Caccamo et al., 2011). This finding underscores the intricate relationship between Aβ and mTOR activation (Tiwari et al., 2019; Kepp et al., 2023) (Extensively Reviewed in (Z. Cai et al., 2015; Mueed et al., 2018)) to pose a self-perpetuating Aβ production cycles in Alzheimer’s disease pathology.

However, it is worth noting that a few studies have reported contrasting findings. Shi and colleagues observed boosted Aβ clearance in mice of both sexes, after mTOR activation caused by the selective loss of microglial Tsc1, a negative regulator of mTOR (Shi et al., 2022). These physiological changes were accompanied by an improvement in cognitive functions in the 5XFAD AD mouse model, suggesting a potential negative correlation between mTOR activation and Aβ plaque burden level (Shi et al., 2022).

#### 3.1.2 Effect of mTOR inhibitors in amyloid-beta pathology

Historical evidence in mice models largely shows that reducing mTOR activity via traditional mTOR inhibitors including rapamycin and rapalogs restores Aβ pathogenesis and cognitive capacities, as summarized in Table 1 (Caccamo et al., 2010; Cassano et al., 2019; Jiang et al., 2014a; Singh et al., 2017b; Van Skike et al., 2021). Spilman et al. first showed that long-term inhibition of mTOR pathway through rapamycin administration (oral, 2.24 mg/kg/day, 13 weeks) lowered Aβ42 levels in transgenic PDAPP(J20) mice, an AD model that overexpresses mutant human APP V717F (Spilman et al., 2010). Furthermore, rapamycin treatment is shown to improve learning and restore spatial memory via the Morris Water Maze (MWM), suggesting that mTOR inhibition blocked or delayed Alzheimer’s symptoms onset in the PDAPP(J20) model. Importantly, these effects were accompanied by autophagy upregulation. Administration of the autophagy inhibitor 3-methyladenine removed the positive effects of rapamycin on Aβ42 levels, in both 3xTg-AD and 7PA2 cells. The outcomes were reciprocated with similar experimental protocols on the Apolipoprotein E ɛ4 (APOE4) transgenic mice as a late-onset AD model (Lin et al., 2017). Recent evidence show that rapamycin not only reduces Aβ level, but also abrogates other pathologies induced by Aβ deposition. Van Skike et al. demonstrated in their experiment on PDAPP(J20) that cerebral blood flow, blood-brain barrier breakdown, and neurovascular uncoupling were partly mitigated via the mTOR-Aβ axis (Van Skike et al., 2018; Van Skike et al., 2021), supporting previous findings (Lin et al., 2017).

**TABLE 1:** Effects of mTOR inhibitor on Aβ pathology.

Model	mTOR inhibitor	Administration	Outcome	References
Animal model				
PDAPP(J20) mice	Rapamycin	Oral administration, 2.24 mg/kg/day, 13 weeks	Reduced Aβ42 level, blocked or delayed AD-like cognitive deficits	Spilman et al. (2010)
Tg2576 mice	Rapamycin	Intraperitoneal injection, (3 mg/kg/day) at 4 months of age for 2 weeks	Rapamycin elevated brain Aβ levels	Zhang et al. (2010)
3xTg-AD mice	Rapamycin	Oral administration, (2.24 mg/kg in chow, 10 weeks)	Rapamycin inhibited mTOR signalling and alleviated Aβ pathology: soluble Aβ40 level was not altered, but Aβ42 levels showed a significant decrease	Caccamo et al. (2010)
3× Tg-AD mice	Rapamycin	Rapamycin (14 mg/kg in chow) was given before or after the formation of Aβ plaques	Prophylactical rapamycin treatment induced autophagy, reduced Aβ, and improved cognition. No changes in AD-like pathology or cognitive abilities were observed when rapamycin was given to mice with established plaques	Majumder et al. (2011)
APP/PS1 mice	Temsirolimus (Rapalog)	i.p. injection, 20 mg/kg every 2 days for 60 days	Temsirolimus elevated Aβ clearance in APP/PS1 mice brain in an autophagy-dependent manner	Jiang et al. (2014a)
PDAPP(J20) mice	Rapamycin	Oral administration, 2.24 mg/kg/day, 16 weeks	Rapamycin partially abrogated learning deficits, improved memory, and reduced Aβ burden	Lin et al. (2013)
APOE4 transgenic mice	Rapamycin	Oral administration, 2.24 mg/kg/day, 6 months	Reduced Aβ, restored cerebrovascular functions, improved learning and memory	Lin et al. (2017)
Intra-hippocampal Aβ injection (I mg/mL) in Wistar rats	Rapamycin	i.p. injection, 0.25 mg/kg b.w./day, 3 weeks	Rapamycin activated autophagy and protected neurons against Aβ-induced synaptic dysfunction	Singh et al. (2017a)
PDAPP(J20) mice	Rapamycin	Oral administration, 14 ppm in chow, 10 months	Rapamycin abrogates brain-blood barrier breakdown	Van Skike et al. (2018)
3× Tg-AD mice	Everolimus (rapalog)	i.c.v. or i.p. injection 0.167 μg/μl/day, 4 weeks	Everolimus reduced human APP/Aβ and tau levels, as well as improved cognitive function	Cassano et al. (2019)
PDAPP(J20) mice	Rapamycin	Oral administration, 14 ppm in chow (estimated 1.65 mg/kg/d), 8 months	Reversed neurovascular uncoupling, reduced Aβ, and improved memory	Van Skike et al. (2021)
5XFAD mice	Rapamycin	Oral administration, 14 ppm active rapamycin (equivalent to 2.24 mg/kg), starting from 4 to 6 months of age for 2–3 months	Rapamycin elevated Aβ oligomer levels in the cortex after 2 months and 3 months treatment. No effect on hippocampus Aβ levels	Shi et al. (2022)
5XFAD mice	Rapamycin	Oral administration, 2.24 mg/kg/day, from 3.5 to 7 months of age	Rapamycin-induced inhibition of mTOR pathway following kindled seizures ameliorated memory deficits but had no effect on Aβ levels	Gourmaud et al. (2022)
Cellular model				
N2a transfected with human “Swedish” APP mutant	Rapamycin	12 h treatment with 0.32 μM to 5 μM of rapamycin	Rapamycin increased total Aβ1-40,42 quantities in 0.64–5 μM of rapamycin	Zhang et al. (2010)
HEK293-APP695	Temsirolimus	24 h treatment with 100 nM of temsirolimus	Temsirolimus boosted Aβ clearance in cell cultures by activating autophagy	Jiang et al. (2014b)
Human Neuroblastoma SH-SY5Y Cells	Rapamycin	1.25 μM of aggregated Aβ were used to intoxicate the SH-SY5Y cells. The cell culture then received 200 nM of rapamycin and were incubated for 24 h	Rapamycin-induced autophagy activation alleviated Aβ-induced oxidative stress, apoptosis and neurotoxicity	Singh et al. (2017b)

Cell culture studies substantiate the role of mTOR inhibitors in mitigating Aβ pathogenesis. Jiang et al. reported that temsirolimus facilitated the activation of autophagy in cells that overexpressed human APP (HEK293-APP695). This activation was evident from changes in biomarkers, with P62 reduced by 55% and microtubule-associated protein one light chain 3 (LC3)-II increased 2.1-fold, respectively. This autophagic activation is responsible for reducing the Aβ42 levels; Aβ42 levels remained unchanged when Atg5, an essential component of the autophagic pathway, was knocked down (T. Jiang et al., 2014a). Singh et al. reported similar results in the Aβ-intoxicated SH-SY5Y neuronal cell line, demonstrating that mTOR inhibition using rapamycin alleviates Aβ-induced oxidative stress, apoptosis, and neurotoxicity in an autophagy-dependent manner (Singh et al., 2017a).

However, few reports had reached inconsistent conclusions (Carosi and Sargeant, 2023; Gourmaud et al., 2022; J. H; Lee et al., 2022; Shi et al., 2022) which calls for caution in clinical translation. Zhang et al. describe rapamycin treatment in APP-transfected murine neuronal like cells (N2a) and Tg2576 mice models to increase Aβ accumulation (Zhang et al., 2010). Other studies on the five times familial AD (5XFAD) mouse model show that although rapamycin may attenuate memory deficits in five times familial AD mouse model (5XFAD) with seizure-induced exacerbation, treatment did not alter, or even increased, hippocampal Aβ42 levels (Singh et al., 2017a; Shi et al., 2022). A possible explanation suggested to this disparity between models is their relative lysosome capacities (Carosi and Sargeant, 2023; Yuan et al., 2023). For instance, 5XFAD has less lysosomal degradation potential due to its mutant PSEN1 gene, which is known to interfere with the lysosomal biogenesis (Carosi and Sargeant, 2023; J. H; Lee et al., 2022). Faulty lysosomal degradation in 5XFAD and Tg2576 AD mouse models recently received support showing reduced lysosomal acidification, intra-lysosomal Aβ buildup, and cell death (J. H. Lee et al., 2022). Thus, it is reasonable that increasing autophagy flux via mTOR inhibitors may worsen Aβ response, although direct evidence has yet been demonstrated. The efficacy of mTOR inhibitor treatment in addressing Alzheimer’s disease (AD) pathology could also exhibit a significant temporal dependence, as illustrated by recent research findings. Specifically, Majumder and colleagues have shown that rapamycin effectively mitigates the accumulation of amyloid-beta (Aβ) plaques and tau tangles in the 3xTg-AD mouse model, but only if administered prior to the formation of these pathological features. In contrast, research by Lin et al. on the PDAPP(J20) mouse model suggests that inhibiting mTOR activity can arrest the progression of AD-like symptoms, diminish amyloid burden, and enhance cognitive function, even when the treatment is initiated post-disease onset (Lin et al., 2013). The observed discrepancies could be attributed to the genetic mutations present in various mouse models, presenting challenges in directly translating findings to human AD patients. Regrettably, this factor has not been adequately considered in the growing body of pre-clinical research that utilizes mTOR-associated inhibitors for Alzheimer’s disease treatment.

### 3.2 mTOR inhibition in tau pathology

#### 3.2.1 mTOR in tau hyperphosphorylation

AD also entails the hyperphosphorylation and aggregation of tau, which is another significant hallmark of the disease. Under normal circumstances, tau is a soluble microtubule-associated protein that assists in microtubule formation and cytoskeleton stability. Tau hyperphosphorylation in AD forms insoluble tau structured termed paired helical filaments and NFTs. Diverse pathogenic effects are mediated by hyperphosphorylated tau, including inability for microtubule binding and microtubule network disassembly, aberrant signalling (Z. Cai et al., 2015), and protein aggregate accumulation (Ossenkoppele et al., 2022) to contribute to neuron death (Z. Cai et al., 2015).

AD patients that exhibit tauopathies often show mTOR activation. mTOR phosphorylation at Ser2448 and Ser2481 have been observed to colocalize with taupathy in the hippocampus and temporal cortex of AD patients (Griffin et al., 2005; J. H; Lee et al., 2022; X; Li et al., 2005). Moreover, mTOR effectors including GSK3β, S6K1, and 4E-BP1 also demonstrate increased phosphorylation at mTOR phosphorylation sites. Human genetic backgrounds that show activating mutations in the mTOR signalling pathway are also significantly more likely to develop tau hyperphosphorylation and AD (H. Y. Cai et al., 2023).

**FIGURE 1:**
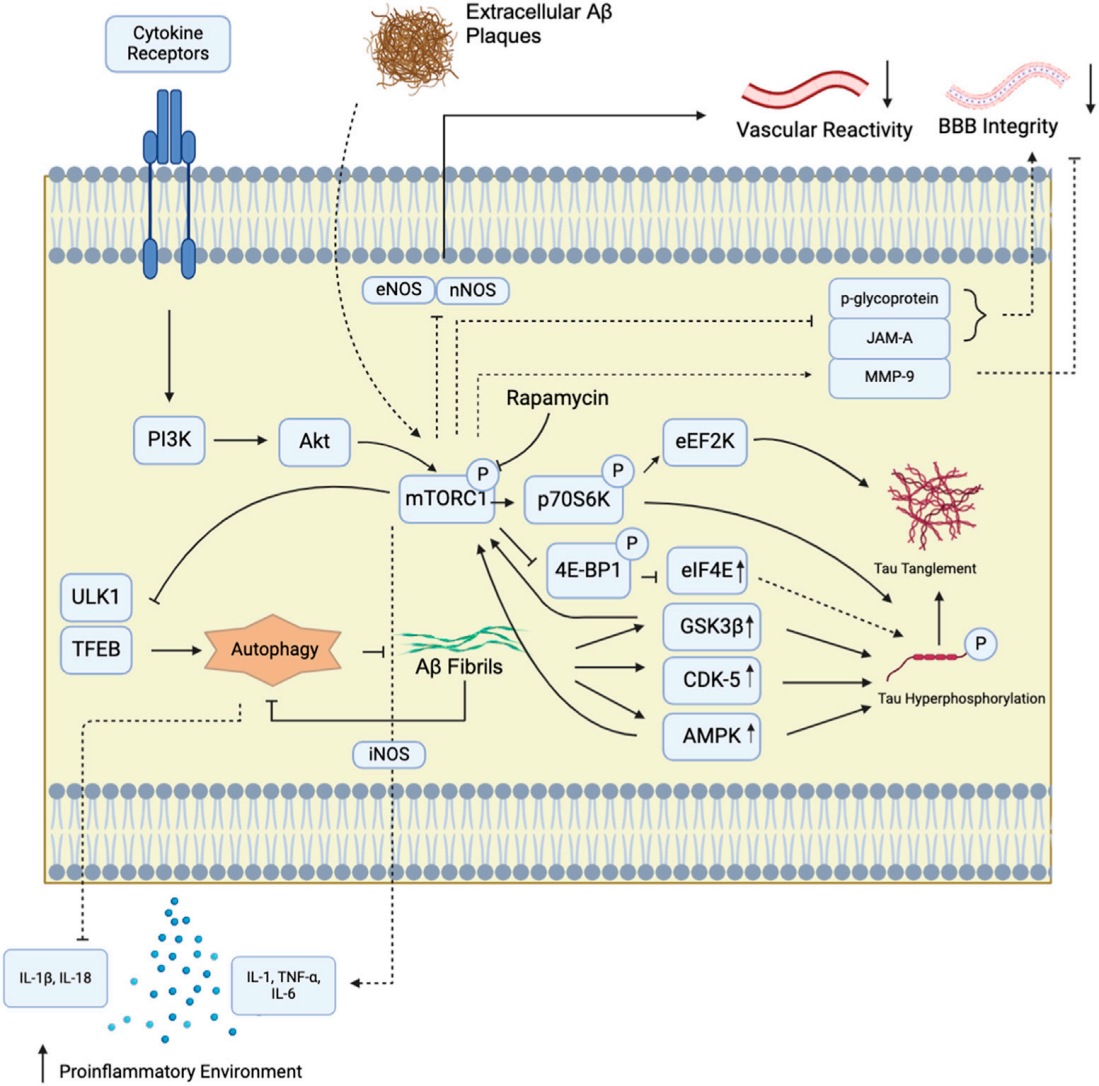
mTORC1 signaling pathway and Alzheimer’s Disease. mTORC1 in brain tissues can be activated by diverse extracellular signals that communicate through cytokine receptors. These receptors switch on the downstream Pi3K/Akt signalling pathway to activate mTORC1. mTORC1 kinase activation has been implicated in numerous AD-associated pathologies. In particular, mTORC1 inhibition has been shown to suppress AD hallmark amyloid-beta (Aβ) production by upregulating autophagy through unc-51-like kinase (ULK1) and transcription factor EB (TFEB) at the lysosome. It also reduces tau hyperphosphorylation and plaque formation by decreasing tau kinase (p70S6K, eEF2K) and protein synthesis (4E-BP1) activities. Tau tanglement can also be enhanced by Aβ aggregation, which accelerate tau phosphorylation via glycogen synthase kinase 3 beta (GSK3β) and cyclin-dependent kinase-5 (CDK-5). In turn, GSK3β and CDK-5 can further activate mTORC1. mTORC1 also indirectly modulates cytokine secretion, upregulates proinflammatory cytokines including IL-1, TNFα, and IL-6. In addition, the mTOR kinase, through reports employing rapamycin, also trigger inducible nitric oxide synthase activation (iNOS), another driver of inflammatory cytokine synthesis. Moreover, autophagy suppression by mTORC1 further suppresses anti-inflammatory signals, including IL-1β and IL-18, to establish neuroinflammation. Disruption of bloodstream-mediated Αβ removal also involves mTOR kinase activity, possibly via mTORC1, to suppress endothelial nitric oxide synthase (eNOS) and neuronal nitric oxide synthase (nNOS) activities to limit vascular responsiveness and waste exchange. In the cerebral vasculature, rapamycin inhibition showed that the mTOR kinase reduces tight junction protein JAM-A and the xenobiotic modulator protein p-glycoprotein levels, and elevates the tight junction breakdown protein matrix metalloproteinase-9 (MMP-9) levels. These interactions are thought to further contribute to the blood-brain barrier (BBB) breakdown in Alzheimer’s patients through defective substrate exchange regulation.

Similar to mTOR promoting Aβ oligomer formation, mTOR interacts with and integrates signalling pathways to propagate tau hyperphosphorylation and aberrant neurofilament formation (Extensively reviewed in (Mueed et al., 2018)). In brief, mTOR does not appears to directly phosphorylate tau but promote tauopathy via increasing tau-protein kinase activity and reducing its autophagic clearance. mTOR has been described to be upregulated by GSK3β, PI3K/Akt axis, insulin/IGF-1, and AMPK, often dysregulated in AD. This suppresses bulk autophagy clearance, and moreover, enhance Aβ-mediated tau hyperphosphorylation via tau kinases and tau CASP3 cleavage (X. Li et al., 2005). Corresponding to the feedback nature of mTOR and Aβ interactions, recent report show that intracellular tau accumulation also activates mTOR to further suppress autophagosome formation (M. Z. Li et al., 2022). Downstream targets of mTOR 4E-BP1 and S6K accelerate microtubule detachment of tau. S6K directly phosphorylates tau; 4E-BP1 release is suggested to mediate differential mRNA translation by eIF4E to impact tau (Ghosh et al., 2020). Indeed, mTOR upregulation in human SH-SY5Y significantly upregulated intracellular tau deposition in diverse cellular compartments (Tang et al., 2015). Interestingly, Tang et al. also showed that mTOR may also play a direct role in the subcellular localization of tau; mTOR mediated tau localization to exocytic vesicles. Lastly, interactions among mTOR, AMPK, and GSK3β also directly elevate tau phosphorylation at serine and threonine residues, hence promoting NFT formation. The roles of the mTOR kinase in Aβ oligomer formation and tauopathies are summarized in Figure 1.

#### 3.2.2 Effect of mTOR inhibitors in tau pathologies

Given these findings, the hypothesis that emerges is that inhibiting mTOR could slow down tau pathology progression in AD (Caccamo et al., 2010). Rapamycin, rapalogs and dual-mTORC1/2 blockers have all exhibited promising results in mitigating tau pathology across a variety of experimental models, as summarized in Table 2 (Caccamo et al., 2010; Frederick et al., 2015; T; Jiang et al., 2014a; Salama et al., 2018). For instance, the inclusion of 2.24 mg/kg rapamycin in the mice diet restored mTOR levels and alleviated tau pathology in 3xTg-AD mice (Caccamo et al., 2010). Rapamycin also reduced tau phosphorylation in rTg4510 mice when injected intraperitoneally (Morawe et al., 2022). It has been suggested that similar to pathological Aβ accumulation, tau pathology in AD is similarly temporally sensitive (Berron et al., 2021). Frederick et al. reported that both prophelactic and therapeutic temsirolimus treatment ameliorate taupathy in 3xTg-mice (Frederick et al., 2015). Rapamycin was administered at five or 10 months, with motor defect onset typically occurring at 8 months. Although total insoluble tau and phosphorylated tau concentrations were lowered in both treatments, NFT density in was only lowered upon prophylactic treatment at 5 months.

**TABLE 2:** Effects of mTOR inhibitor on tau pathology.

Model	mTOR drug	Administration	Outcome	References
Animal models				
3xTg-AD mice	Rapamycin	Oral administration, (2.24 mg/kg in chow, 10 weeks)	Rapamycin restored mTOR level and attenuate tau pathology	Caccamo et al. (2010)
3xTg-AD mice	Rapamycin	Rapamycin (14 mg/kg in chow) was given before or after the formation of Aβ plaques	Tau aggregation was reduced when rapamycin was given prophylactically, but not after plaques and tangles were already established	Majumder et al. (2011)
P301 Tau Transgenic mice	Rapamycin	15 mg/kg administered intraperitoneally twice per week from 3 weeks to 5.5 months of age or from 3 months to 4.5 months of age	Reduction in tau tangles, tau hyperphosphorylation, lowered insoluble tau levels. Qualitative reduction in astrogliosis. Observed in both early preventative and late treatment	Ozcelik et al. (2013)
CD1 mice	Rapamycin	CD1 mice were injected with AAV-hTauP301L, carrying full-length human tau with 4 repeats and the P301L mutation	Rapamycin suppressed tau-induced neuronal loss, synaptotoxicity and gliosis, but did not alter human tau mRNA or protein levels	Siman et al. (2015)
Tg (Thy1-MAPT*)30Schd mice	Temsirolimus	Transgenic mice received 200 μg/kg body weight i.p. injection for 3 time per week, either (A) from 10–12 month of age (after motor deficit onset), or (B) from 5–12 month of age (prior to motor deficit onset)	Temsirolimus reduced the levels of insoluble tau and phosphorylated tau in both group	Frederick et al. (2015)
hTau.P301S transgenic mice	Temsirolimus	20 mg/kg temsirolimus were i.p. injected every 2 days for 60 days	Temsirolimus reduced hyperphosphorylated tau level and improved spatial cognitive impairments	Jiang et al. (2014a)
hTau.P301S mice, rTg4510 mice	Rapamycin and PQR530	hTau.P310S mice: rapamycin was injected 15 mg/kg i.p., twice per week from 2–5 months or from 3.5–5 months. PQR530 was injected at 30 mg/kg i.p. twice per week from 3.5–5.5 months. rTg4510 mice: rapamycin was injected 15 mg/kg i.p. twice per week from 2.5–5.5 months	Rapamycin reduced tau hyperphosphorylation and aggregation in cerebral cortex when administered to young hTau.P310S and rTg4510 mice only. PQR530 attenuated tau pathologies when administered to older mice	Morawe et al. (2022)
Sprague–Dawley (SD) rats laterally ventricularly injected with 25 mM, 2 μL of zinc sulphate solution	Rapamycin	1.5 mg/kg injected i.p. three times for 1 week post zinc administration	Reduced tau phosphorylation, oxidative stress, synaptic impairment, and spatial cognition loss	Lai et al. (2022)
Cellular models				
HEK293/tau441	Rapamycin	HEK293 were transfected with human tau (tau441). Treated with 1 uM rapamycin for 2 h	Reduced Tau ser214 phosphorylation	Liu et al. (2013)
SH-SY5Y cell line	Temsirolimus	Human SH-SY5Y neuroblastoma cells were incubated with 5 nM okadaic acid for 4 h, to establish tauopathy model. The cells were then treated with 100 nM temsirolimus for 24 h	Phosphorylated tau/tau ratio was decreased by 58%, whereas the total tau remained unchanged. Further analysis indicated that autophagy induction and GSK-3β inactivation contributed to this change	Jiang et al. (2014b)
LUHMES (Lund human mesencephalic) cell line	PP242	Tauopathy was established in cells by adding 64 nM fenazaquin to the culture medium. 1 uM PP242 was also added to the cell in the experimental group	PP242 attenuated fenazaquin-induced tauopathy, reducing the percentage of neurons with positive Tau aggregates from 37% to 5%	Salama et al. (2018)
iPSC-derived neuronal cell line carrying tau-A152T or tau-P201L mutation)	OSI-027; AZD 2014; AZD8055; rapamycin	0.1–10 μM, 24 h	mTOR inhibitor administration promoted tau reduction. OSI-027, AZD 2014, and AZD8055 did not display neurotoxicity at concentrations≤30 µM	Silva et al. (2020)

Studies on P301S tautransgenic mice largely support that mTOR inhibitors may attenuate tau hyperphosphorylation in AD when administered both therapeutically and prophylactically (T. Jiang et al., 2014b; Morawe et al., 2022; Ozcelik et al., 2013). Ozcelik et al., using the P301S model overexpressing the 0N4R tau isoform under the Thy-1.2 promoter element, observed a decrease in both total tau phosphorylation and tau tangles in the cortex upon rapamycin prior to and after tau hyperphosphorylation onset. Jiang et al., applying an unspecified P301S in the C57Bl/6J background, showed that therapeutic systemic temsirolimus treatment at 5 months reduces tau phosphorylation and improve spatial cognitive impairments. An upregulation of autophagy markers is a consistent theme, along with reduced 4E-BP1, S6K, and GSK3β phosphorylation. Cell line taupathy models substantiated these findings. SH-SY5Y neuroblastoma cells with established tau pathology reported a reduction in phosphorylated tau, autophagy induction, and GSK3β inactivation upon 100 nM temsirolimus treatment (T. Jiang et al., 2014a). In fact, mTOR inhibitors is reported to generally alleviate taupathies in cell models (T. Jiang et al., 2014a; Y; Liu et al., 2013; Silva et al., 2020). Recent evidence indicates that mTOR inhibition can limit tau phosphorylation associated with environmental causes of Alzheimer’s, including zinc (Lai et al., 2022) and aluminum toxicity (Y. Xu et al., 2022). Note that less commonly used mTOR inhibitors can also alleviate tau pathology. PP242, a dual mTORC1/C2 blocker, was reported to effectively reverse fenazaquin-established tauopathy (Salama et al., 2018).

However, a few studies registered inconsistent experimental outcomes. Using the 3xTg-AD mice, Majumder and colleagues only observed reductions in tau aggregation when rapamycin was given as a preventive measure, before the formation of Aβ plaques (Majumder et al., 2011). The effect of rapamycin on downstream proteins was markedly different when a different AD model was applied. Siman et al. established tauopathy model by injecting viral vectors for mutated human tau, containing four repeats and a P301L mutation, into CD1 background mice. The researchers observed that rapamycin suppressed various harmful effects of tau, including neuronal loss, synaptotoxicity, and gliosis, but the levels of human tau mRNA or protein were not altered (Siman et al., 2015). Recent investigation of therapeutic treatment of rapamycin in P301S mice, expressed under the Thy1-promoter, by Morawe et al. show that rapamycin administered therapeutically at 3.5 months does not reduce phospho-tau (T212/S214) levels or total insoluble tau levels (Morawe et al., 2022). It is conceivable that these effects may be due to varying pharmacological properties of mTOR inhibitors. Frederick et al. and Jiang et al. employ temsirolimus, which has greater solubility and delivery capacities pass the blood-brain barrier (BBB) than rapamycin, which Majumder et al. and Morawe et al. use. Morawe et al. show that PQR530, an PI3K/mTORC1 dual inhibitor with significantly greater BBB permeability than rapamycin, does alleviate tauopathy when administered at 3.5 months. Nonetheless, these disparities highlight the model-dependent nature mTOR inhibitors response. Furthermore, few studies evaluate the synergistic role of Aβ plaques in Alzheimer’s tauopathy, further urging clinical caution.

### 3.3 mTOR inhibition in Alzheimer’s disease neuroinflammation

#### 3.3.1 Neuroinflammation and Alzheimer’s disease

As in many neurodegenerative conditions, neuroinflammation represents a fundamental characteristic of Alzheimer’s disease. The activation of proinflammatory signalling cascades results in the release of immune mediators that profoundly affect neuronal functions, often culminating in cell death. Neuroinflammation has a dual impact on the brain. On one hand, it aids in clearing deposited Aβ, while on the other hand, it produces cytotoxic substances that exacerbate Aβ deposition and contribute to neurodegeneration. Numerous experiments have provided evidence of the high accumulation of inflammatory mediators around NFTs and amyloid plaques, highlighting the significant involvement of neuroinflammation in AD (Atri, 2019). The AD brain observed an increased level of microglia- and astrocytes-released IL-1, which facilitates the abnormal processing of APP and consequently the production of Aβ. Aβ, in turn, is considered to further boost the concentration of interleukins. Interleukins, including IL-4 and IL-10, affect Aβ clearance and inflammation. IL-4 decreases Aβ clearance, leading to the deposition of senile plaques, while IL-10 acts as an anti-inflammatory molecule, suppressing pro-inflammatory mediators. These findings highlight the intricate interplay between interleukins and AD pathology, where cytokine dysregulation contributes to neuroinflammation and neurotoxicity, ultimately demonstrating a clear correlation with AD development and progression.

The role of the mTOR in modulating immune responses is well-established, yet its specific influence on the development of AD through neuroinflammatory pathways is still a subject of ongoing research (Cheng, Wei, Qian, and Han, 2023). A compromised anti-inflammatory response can aggravate neuroinflammation, with several key signalling pathways—including NFκB, p38 MAPK, Akt/mTOR, nitric oxide, and COX—playing critical roles in driving immune reactions. Ko et al. found that the enhancement of autophagy via rapamycin treatment can inhibit the secretion of proinflammatory cytokines IL-1β and IL-18. This decrease in IL-1β correlates with a reduction in the activation of the p38 MAPK-NFκB pathway and a consequent downregulation of inflammatory agents like IL-6, IL-8, MCP-1, and IκBα in macrophages treated with rapamycin (Bagyinszky et al., 2017; Ko et al., 2017).

Moreover, the involvement of brain immune cells like astrocytes and microglia is significant, as their dysfunction is often linked to neurodegenerative conditions (Thakur et al., 2023). Microglia, as the resident macrophages and primary immune cells of the CNS, are essential in modulating inflammation and ensuring neuroprotection. Ye et al. found that lipopolysaccharide-induced inflammation reduces autophagic activity in N9 microglial cells, indicated by lower levels of the autophagy markers LC3-II and SQSTM1. Administration of the autophagy inducer, rapamycin, however, decreased the transcript level of pro-inflammatory cytokines, including TNF-α, IL-6, and iNOS (Ye et al., 2020). Similarly, astrocytes also facilitate normal brain function in a mTOR-dependent manner. Li et al. showed that by inhibiting mTOR signal pathway with rapamycin for 24 h, in astrocytes obtained from rat cerebral cortices, it suppressed oxygen-glucose deprivation/reoxygenation-induced astrocytic proliferation and migration. Concomitantly, following rapamycin administration, the expression of inflammatory mediators such as TNF-α and iNOS were reduced relative to control (C. Y. Li et al., 2015). This evidence collectively underscores the intricate interplay between mTOR signalling, glial cell regulation, and the neuroinflammatory landscape in AD.

#### 3.3.2 mTOR inhibitors in AD-associated neuroinflammation

**FIGURE 2:**
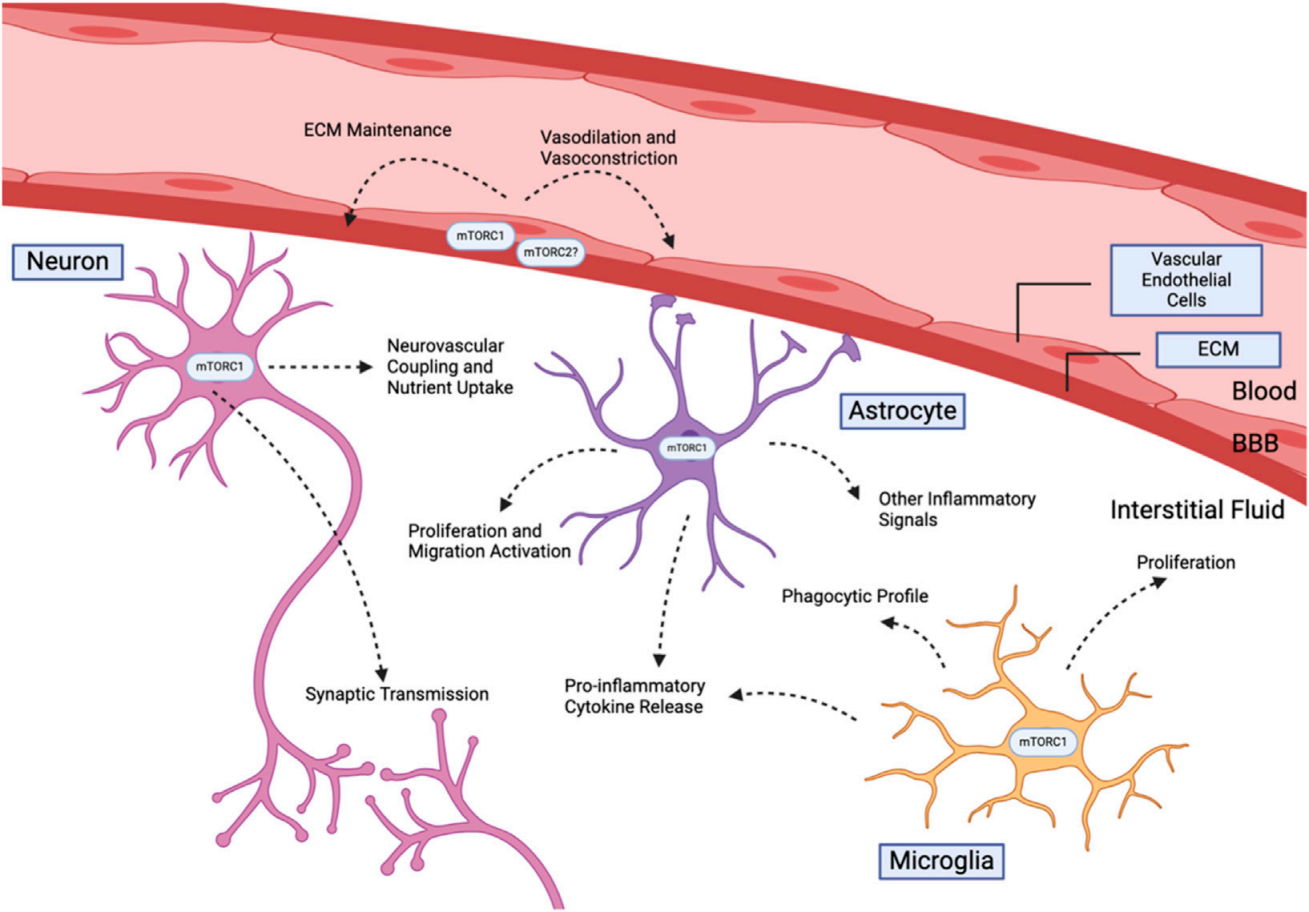
Summary of key cerebral cell types that are modulated by mTOR kinases.

In the context of Alzheimer’s models, mTOR inhibition has generally been associated with reduced brain inflammation and reduced glia activation. Figure 2 summarizes the known functions of the mTOR complexes in the brain. Oral administration of rapamycin to APOE4 transgenic mice starting at 1 month displayed reduced proinflammatory cyclophilin A (Cyp4) levels and cognitive function improvements (Lin et al., 2017). Inflammation induced by Aβ exposure may also be resolved by mTOR inhibition. Injection of Aβ25-35 into the hippocampus of C57BL/6 mice increased *in vivo* astrocyte proliferation, while simultaneous rapamycin and Aβ25-35 injection show significantly reduced proliferation (Y. C. Liu et al., 2017). At the same time, rapamycin administration reduced pro-inflammatory signalling markers, including NF-κBm, IL-1β, and TNF-α levels, of Aβ25-35-infused mice. (Y. C. Liu et al., 2017). These findings are consistent with other studies of mTOR inhibition in response to pro-inflammatory stimulants. Rapamycin reduces the pro-inflammatory environment associated with lipopolysaccaride (LPS) injections, physical damages to nervous tissue, and seizures through limiting various inflammatory cytokine release and astrocyte proliferation (Guo et al., 2017; C. Y; Li et al., 2015; F; Wang et al., 2023; Ye et al., 2020). We note that one study using intracerebroventricular (ICV) infusion demonstrated opposite results (X. Wang et al., 2016). Aβ1-42 injection followed by rapamycin showed an elevated inflammatory profile with increased IL-6 and TNFα levels, accompanied by increased pro-apoptotic caspase 3 activation and worsened spatial working memory ((X. Wang et al., 2016). ICV infusion is an uncommon method of Aβ1-42 and rapamycin delivery that bypasses the blood brain barrier, which may produce difference in effective dosage.

Whether a reduction in neuroinflammation following mTOR inhibition alleviates Alzheimer’s has been subject to mixed evidence. It has been found that reduced inflammation may reduce complement protein activation, free radical generation, cytokine release, and microglia and astrocyte proliferation and phagocytosis. Subsequently, reduced brain oxidative stress follows, which rescues amyloid plaque and tau tangle formation. Moreover, this reduces AD-associated nervous structure changes includes synapse engulfment and pyroptosis (Hong et al., 2016). This line of reasoning is supported by improved learning and memory capacities following anti-inflammatory treatments after LPS challenges or in response to other non-amyloidogenic neuroinflammatory conditions (Davoody et al., 2023).

Recent evidence show that rapamycin may suppress microglia activation and establishment of a microglia phagocytic profile. Shi et al. showed that activating mTOR kinases in the microglia of 5XFAD mice shows enhanced amyloid plaque clearance, improved cognitive functions, and reduced spine loss (Shi et al., 2022). Following rapamycin treatment, it reduced the number of mTOR-activated microglia and worsened amyloid pathology. mTOR activation increased phagocytosis receptor TREM2 expression and lysosomal biogenesis, which became suppressed upon mTOR inhibition (Shi et al., 2022). Reduced mTOR signalling is also associated with how amyloid-beta peptides can suppress microglia phagocytosis over time. Baik et al. show that chronic microglia exposure to Aβ reduces IL-1B and TNF-a expression and phagocytic activity in an AKT-mTOR-HIF1α signalling-dependent pathway (Baik et al., 2019). This desensitized phenotype has been reported to be analogous to the anti-inflammatory and non-Aβ phagocytic microglia profiles after mTOR inhibition, including the J20 model (Pomilio et al., 2020), 5xFAD isolated microglia (Grubman et al., 2021), and has been suggested in astrocytes. It is possible that the extent of Aβ-induced microglial exhaustion could account for the diverse response to rapamycin treatment in different neuroinflammation models.

### 3.4 mTOR inhibition in Alzheimer’s neurovascular dysfunctions

#### 3.4.1 Structure and function of the neurovascular system

The neurovascular system is a highly heterogeneous structure that involves diverse cerebral and microvasculature components (Muoio et al., 2014). This includes neurons, microglia, astrocytes, vascular endothelial cells, extracellular basement membrane, and a plethora of other cellular and acellular structures. They coordinate in response to external signals or local inducers to produce activities in a spatiotemporal specific manner, supporting both normal brain physiology and response to environmental stimuli. Examples include oxygen uptake, selective nutrient and waste exchange, neuronal signal transduction support, hormonal signal diffusion, etc.

A key function of the neurovascular system is responsible to maintain appropriate cerebral blood flow (CBF) quantities to support normal waste-clearance and oxygen nutrient distribution. The brain is well-known to consume 20% of total body energy supply, and requires an extensive oxygen delivery system for metabolism. This has been shown to be mediated by tight vascular cell dilation and constriction control via endothelial nitric oxide and neuronal nitric oxide production (Förstermann and Münzel, 2006). This dynamic signal integration allows for exact control of blood flow quantities in response to stimuli, through a process known as neurovascular coupling. This is responsible for rapid global or localised increases in nutrient uptake following elevated energetic demands from brain tissues. Disruptions in the process are commonly associated with disease (Förstermann and Münzel, 2006). Both reduced CBF (hypoperfusion) and excessive CBF (hyperperfusion) may cause acute ischemia, hypertension, stroke, and neurodegenerative diseases; neurovascular uncoupling is common to various forms of dementia.

Selectivity of nutrients and signals from the brain vasculature is maintained by the blood-brain barrier (BBB). The BBB is a highly complex structure which dynamically regulates molecule exchange between the cerebral interstitial fluid and blood (Reviewed in (Kadry et al., 2020)). Brain microvascular endothelial cells express transporters and junction proteins on the BBB for its activity and specificity. Moreover, recent evidence has demonstrated a key regulatory role by glial cells, notably microglia, astrocytes, and pericytes, in maintaining BBB integrity and function. In neurodegenerative diseases, physical damage to the BBB and protein expression alterations have been described to associated with reduced brain oxygen supply, reduced amyloid plaque clearance, neuroinflammation, neuron death, and gliosis.

#### 3.4.2 mTOR inhibition in Alzheimer’s neurovascular system

Chronic cerebral hypoperfusion (CCH) has been noted to play an important role in the progression of AD through reducing extracellular Aβ plaque clearance, which may in turn further impair neurovascular activities, contributing to a self-sustaining cycle of disease progression. CCH is a key element of the vascular hypothesis, where cardiovascular diseases and amyloid plaque deposits are hypothesized to increase brain local inflammatory signals, elevate oxidative stress, compromise vascular functions, and induce neuronal cell death. Notably, CCH has been hypothesized as a primary mediator of Alzheimer’s progression that precedes tau hyperphosphorylation and amyloid plaque deposition (Park et al., 2019).

Current evidence largely supports mTOR inhibition to improve reduced CBF in AD. Interestingly, as opposed to worsened intracellular Aβ accumulation in late-stage lysosomal dysfunctions, mTOR inhibition has been consistently shown to rescue existing vascular defects and global Aβ levels. Lin et al. show that rapamycin administration at 7 months of age for 16 weeks to h (APP) mice reduced Anti-Ab antibody colocalization with brain vasculature, reduced microhemorrhages, restored CBF, and improved spatial memory (Lin et al., 2013). mTOR inhibition upregulates endothelial nitric oxide synthase (eNOS) to promote vasodilation and Aβ efflux from interstitial fluids (Lin et al., 2013). Notably, this indicates that late stage mTOR inhibition can improve AD pathologies via a non-autophagy mediated mechanism, in disagreement with lysosome-centric viewpoints as the acting mechanisms of mTOR inhibitors. Rapamycin treatment in the E4FAD Alzheimer’s mice model by Bell et al. replicates previous results, displaying increased CBF parameters, attenuated extracellular Aβ formation, and improved learning. The E4FAD model overexpress the human AD susceptibility allele APOE4, which may produce vascular damages years prior AD onset (Bell et al., 2012). Bell et al. administered rapamycin post-vascular defect onset, suggesting that mTOR inhibition by rapamycin may rescue both existing vascular defects and the following AB deposits. This may represent a body-wide improvement as opposed to only the cerebral vasculature. Rapamycin has also been recently shown to mitigate peripheral blood flow loss in h (APP) mice (Van Skike et al., 2023).

CBF restoration also leads to neurovascular uncoupling rescue. Neurovascular uncoupling occurs in Alzheimer’s patients where CBF does not increased in response to high neuronal activity. This cause oxygen and nutrient deprivation to directly cause neuron death and gliosis. Van Skike et al. fed rapamycin-supplemented chow to PDAPP(J20) mice at effectively 1.65 mg/kg weight, from 2 or 8 months to 10 or 12 months of age respectively (Van Skike et al., 2021). The control model displays significant loss in CBF increase upon whisker stimulation, indicating compromised neurovascular coupling. Both early and late rapamycin feeding, before and after the onset of neurovascular uncoupling, rescued this CBF reduction, reduced Aβ42 accumulation in the somatosensory cortex, and improved contextual memory following contextual fear conditioning (Van Skike et al., 2021). This temporally independent characteristic has been suggested to be due to the intact lysosomal system specific to J20 mice (Carosi and Sargeant, 2023). Distinct from their previous paper by Lin et al., the group demonstrates that this improvement is primarily mediated by mTOR elevation of neuronal NOS (nNOS) activity. When blocked with either the nNOS specific inhibitor L-NPA or general NOS inhibitor L-NAME, it erases the associated CBF improvements (Lin et al., 2013).

Recent findings by Jiang et al. show that rapamycin partially attenuates LRP-1 downregulation in human brain microvascular endothelial cell (HBMECs) models under high glucose conditions (G. Jiang et al., 2023). This reduces SREBP1 nuclear translocation and β-secretases expression, limiting Aβ synthesis. Rapamycin treatment in HBMECs and cerebrovascular endothelial cell *raptor* silenced mice both displayed increased Aβ efflux rates, and improved mice cognitive capabilities (G. Jiang et al., 2023). mTOR inhibition also reduces normal age-associated in microvasculature density declines and hypoperfusion, independent of Aβ (Van Skike et al., 2020). Of note, none of the studies described investigates the clearance of extracellular tau-tangles by mTOR inhibition’s effects on CBF. Emerging evidence from Hussong et al. show that taupathy blocks eNOS activation and induce vascular cell senescence (Hussong et al., 2023), which mTOR attenuation may effectively rescue given its overlapping role in both processes.

In BBB dysfunctions, current evidence largely points at mTOR inhibitors to restore brain BMEC protein expression changes associated with AD. In an h (APP)J20 mice model, rapamycin feeding at 5 months of age for 10 months reduced extravasated dextran levels in brain interstitial fluids and upregulated cell adhesion protein JAM-A (Van Skike et al., 2018). Rapamycin also downregulates matrix metalloproteinase-9 (MMP-9), an enzyme implicated in tight junction protein breakdown. The authors describe that mTOR inhibitors ameliorate BBB breakdown via restoring tight junction expression. However, the group did not find other BMEC tight junction proteins expression including claudin-5 or zonula occludens 1 (ZO-1) to change (Van Skike et al., 2018), suggestive of functional variations of tight junction proteins in AD pathology. mTOR inhibition also restores local vasculature homeostasis via BMEC efflux transporter upregulation. E4FAD mice following rapamycin treatment recovered p-glycoprotein levels to E3FAD control levels (Lin et al., 2020). P-glycoprotein is required for removal of toxic substances and xenobiotics, which become compromised in AD. We did not yet identify studies describing microglial inflammatory profiles in BBB maintenance and BMEC expression profiles under mTOR inhibition in AD, despite abundant associations of junction disassembly and global BBB integrity with microglia inflammatory states (You et al., 2016; Haruwaka et al., 2019; Huang et al., 2023), a process known to be controlled by mTOR.

## 4 Conclusion and perspectives

**FIGURE 3:**
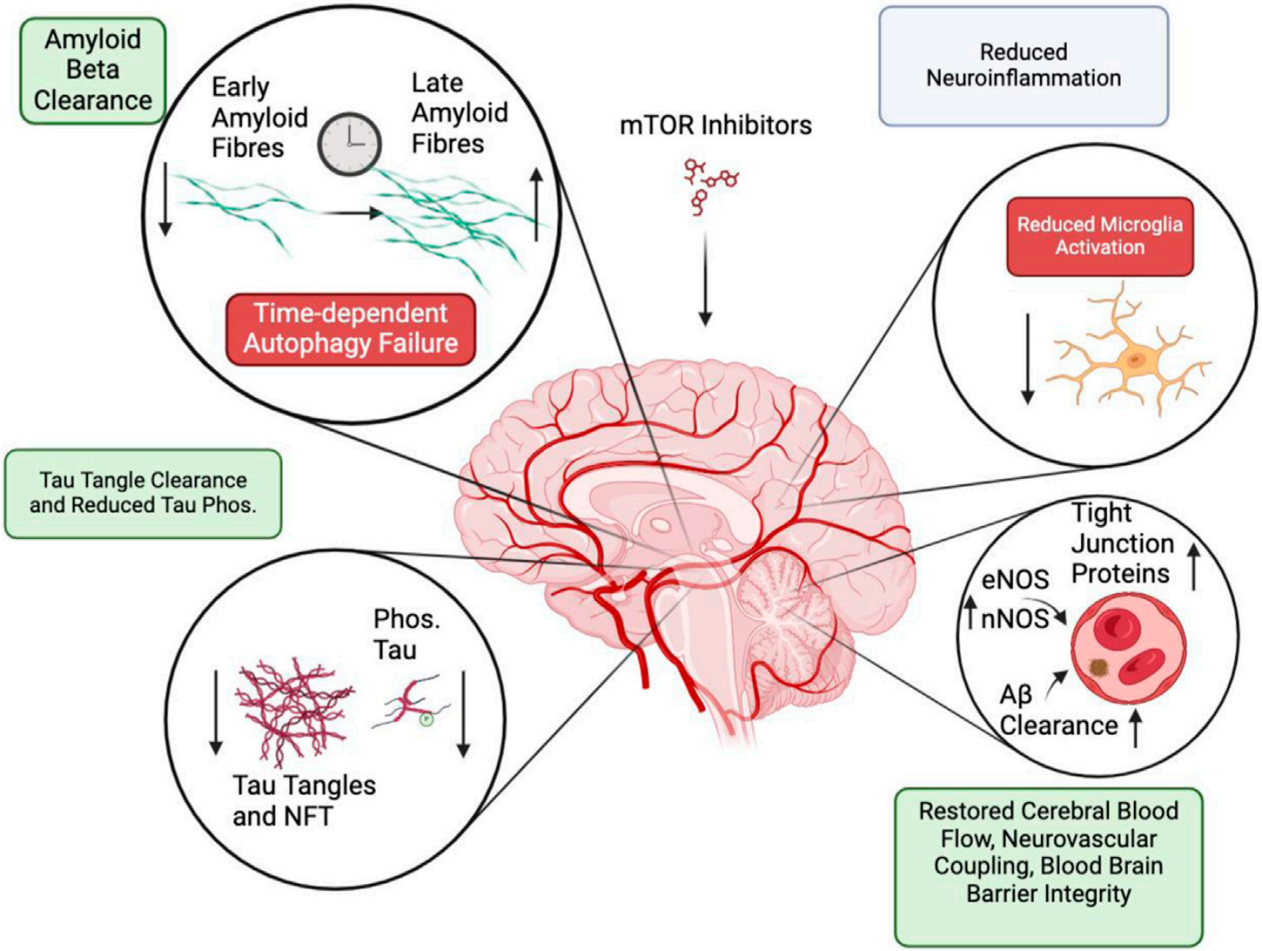
Summary of processes that are modulated by mTOR inhibition. Positive effects (Green) are displayed in Amyloid-beta clearance, tau tangle phosphorylation and clearance. Potentially negative effects (Red) include autophagy failure and reduced microglia activation. Mixed effect (Grey) are found following neuroinflammation resolution.

mTOR inhibition modulates a plethora of pathologies associated with AD. In sum, it is implicated in successfully elevating the clearance of Aβ fibrils and aggregates through autophagy and elevated blood flow, reduced tau hyperphosphorylation and tau filament removal, lowered neuroinflammation, and blood-brain barrier restoration (Figure 3). However, these successes must be taken with a grain of salt. mTOR inhibitor’s role in Aβ clearance is likely dependent on the time of administration, where a progressed disease state could elevate the level of lysosomal dysfunctions, which makes mTOR inhibitor’s elevated autophagy ineffective. Alternatively, AD models with an intrinsic lysosomal deficiency may also not response to mTOR inhibition intervention. Improved knowledge of the AD etiology concerning neuroinflammation is also necessary. It remains uncertain how microglia and astrocyte proliferation modulate neuronal death and cerebrospinal Aβ aggregates. Experiments that compare mTOR intervention outcomes before, and after, the onset of pathological markers will benefit treatment. This should be conducted in diverse model systems to avoid modelling biases; mice AD models typically have only a few types of AD pathologies. Better cell-type specific understanding of mTOR inhibition functions would clarify the varied outcomes between brain endothelial cells, glial cells, and neurons. In addition, an exploration of non-rapamycin mTOR inhibitors may be useful; current literature revolves around rapamycin when assessing the role of mTOR complexes in AD. These considerations will complement the upcoming findings from the clinical trials administrating rapamycin to MCI Alzheimer’s patients.
